# MASTering systemic mastocytosis: Lessons learned from a large patient cohort

**DOI:** 10.1016/j.jacig.2024.100316

**Published:** 2024-07-27

**Authors:** Kevin Y. Tse, Wansu Chen, Eric J. Puttock, Shanta Chowdhury, Kerri Miller, Dakota Powell, Benjamin Lampson, Chris Yuen, Doug Cattie, Teresa Green, Erin Sullivan, Robert S. Zeiger

**Affiliations:** a Department of Allergy, Kaiser Permanente Medical Center, San Diego, Calif; b Department of Research and Evaluation, Kaiser Permanente Medical Center, Pasadena, Calif; c Blueprint Medicines Corporation, Cambridge, Mass; d Department of Clinical Science, Kaiser Permanente Bernard J. Tyson School of Medicine, San Diego, Calif

**Keywords:** Mastocytosis, diagnostic delay, health care utilization, progression, mortality

## Abstract

**Background:**

Systemic mastocytosis (SM), a rare condition affecting about 32,000 individuals in the United States, is often misdiagnosed or underdiagnosed owing to its nonspecific symptoms and the need for invasive biopsies.

**Objective:**

Our aim was to identify, classify, and characterize the natural history of patients with SM.

**Methods:**

In a retrospective cohort study, administrative data from a large managed care organization was used to identify patients with confirmed SM, based on World Health Organization criteria. Demographic data, delay to diagnosis, disease progression, and health care resource utilization were examined.

**Results:**

Of 116 patients with confirmed SM, 77% had indolent SM, 2% had smoldering SM, 12% had SM with associated hematologic neoplasm, 9% had aggressive SM, and none had mast cell leukemia. In all, 5 patients were misclassified as having a less advanced SM subtype initially and 3 were completely undiagnosed (missed diagnosis). The average delay to diagnosis of SM was 58.3 plus or minus 73.1 months. In all, 18% of patients progressed from a nonadvanced form of SM (indolent or smoldering SM) to an advanced form of SM (aggressive SM, SM with associated hematologic neoplasm, or mast cell leukemia) over an average of 88.3 plus or minus 82.7 months. Patients with SM had increased health care utilization, including increases in their numbers of hospital admissions, emergency room visits, urgent care visits, and specialty provider visits, after diagnosis versus before.

**Conclusions:**

Rare diseases such as SM would benefit from increased understanding and awareness to improve diagnostic accuracy. Prospective studies are needed to better characterize this patient population and determine the type of follow-up needed to recognize advanced forms of SM so that appropriate treatment can be implemented.

Systemic mastocytosis (SM) is a rare mast cell disease affecting an estimated 1 in every 10,000 to 200,000 individuals.^1^ It is estimated that there are 32,000 patients with SM in the United States.^2^ SM is characterized by the proliferation of aberrant mast cells in multiple extracutaneous and cutaneous tissues, which can cause significant symptom burden and poor quality of life. In fact, patients with SM often experience debilitating symptoms, including (but not limited to) cutaneous symptoms (flushing, itching, and hives), gastrointestinal symptoms (abdominal pain, diarrhea, and nausea and/or vomiting), neuropsychiatric symptoms (depression, mood changes, and “brain fog”), musculoskeletal disorders (bone and muscle pain and osteoporosis), and life-threatening anaphylaxis. These symptoms can occur in all cases of SM, including the most common type, which is termed *indolent SM* (ISM). In advanced SM, symptoms can include lymphadenopathy, hepatosplenomegaly, pancytopenia, lytic bone lesions, and pathologic fractures, and they can be associated with various hematologic disorders. These advanced cases are termed *SM with associated hematologic neoplasm* (SM-AHN), *aggressive SM* (ASM), and *mast cell leukemia* (MCL).

The diagnosis of SM and its subtypes is aided by 2 separate international governing societies that provide clear guidelines, namely, the World Health Organization (WHO)^3^^,^^4^ and the International Consensus Classification.^5^

However, some important characteristics of the disease, including the risk of progression from ISM to more advanced forms of SM, can be a challenge even when following the aforementioned guidelines. Additionally, there is often a relative lack of understanding or awareness of SM, frequently contributing to missed diagnosis, misdiagnosis, and delays in diagnosis.

SM is driven primarily by a gain-of-function mutation (*D816V*) in the *c-KIT* gene,^6^ which can be detected in bone marrow biopsy samples, as well as in peripheral blood (although this can be somewhat dependent on level of mast cell burden). Wild-type *c-KIT* is a tyrosine kinase receptor that requires its ligand, stem cell factor, for activation. However, the *D816V* mutation is an activating mutation that has been strongly linked to development of SM (present in 95% of SM cases), yet it is considered only a minor criterion for diagnosing SM according to both the WHO and International Consensus Classification guidelines.^2^^,^^4^^,^^5^^,^^7^ Other relevant diagnostic factors include serum tryptase levels; bone marrow biopsy findings of multifocal aggregates of mast cells (>15/hpf per aggregate); atypically shaped mast cells; and abnormal coexpression of non–mast cell surface markers, including CD2, CD25, and CD30.^8^^,^^9^

Kaiser Permanente Southern California (KPSC) is one of the nation’s largest health maintenance organizations, with access to millions of patient records and the ability to comprehensively capture significant amounts of health care information related to signs and symptoms, testing and imaging results, health care resource utilization, and pharmacy dispensing. The KPSC Research Data Warehouse^10^ provides researchers with a wealth of clinical details, which allows them to determine the frequency of SM among its members via physician chart review of patients who were suspected of having SM between the years of 2008 and 2023 according to the WHO diagnostic criteria available at the time of initiation of this study.^3^^,^^4^ The purpose of the present study was to retrospectively identify patients with SM and determine the rates of disease progression (from ISM to various advanced forms), health care resource utilization, referral patterns, and time to diagnosis. We hypothesized that the large-scale, patient-level information available within the detailed KPSC medical record system could allow us to determine the approach to SM diagnosis and determine health care resource utilization in a sizeable cohort of patients with SM in a real-world setting.

## Methods

### Identification of eligible patients with SM and their subtypes

In this retrospective cohort study, the electronic medical records (EMRs) of patients aged 18 years or older within the KPSC health system were used to identify patients with at least 1 *International Classification of Diseases, Ninth Revision* or *10th Revision* code for SM (see the Appendix in the Online Repository at www.jaci-global.org). Patients were manually confirmed as having SM by physician review of each individual medical record. The WHO 2016 criteria were used because at the time of initiation of this study, the WHO 2022 update had not yet been published. Patients with only partial or incomplete data were confirmed on the basis of the best available historical data. Three subjects were identified solely by bone marrow report, without a physician diagnosis (see Fig E1 in the Online Repository at www.jacig-global.org).

### SM subtyping

SM subtypes were determined on the basis of established criteria for subclassification (according to the 2016 WHO guidelines^3^ [Table I]).

**Table I:** Systemic mastocytosis subtypes based on 2016 WHO guidelines

SM subtype	Definition
ISM	Patient meets the WHO criteria for SM (1 major and ≥1 minor criteria OR ≥3 minor criteria) but does not exhibit any “B” or “C” findings, does not have an associated hematologic malignancy, and should have <20% mast cells in the bone marrow aspirate. ISM is the most prevalent form of SM
SSM	SM is distinguished from ISM by the presence of ≥2 “B” findings, which include 1. Bone marrow biopsy showing >30% infiltration by mast cells (dense, focal aggregates) and serum tryptase level > 200 mg/mL 2. Signs of dysplasia or myeloproliferation in non–mast cell lineages (but insufficient to be considered SM-AHN, see later) 3. Hepatomegaly (without impairment of liver function) and/or palpable splenomegaly without hypersplenism or lymphadenopathy on palpation or imaging
ASM	SM with no associated hematologic neoplasm, and <20% mast cells in the bone marrow but with ≥1 “C” finding (ie, organ function impairment due to excessive mast cell infiltration) based on the following criteria: 1. Bone marrow dysfunction resulting in ≥1 cytopenias (absolute neutrophil count of <1 × 10^9^/L, anemia with hemoglobin level of <10 g/dL, or platelet count of <100 × 10^9^/L) without an obvious non–mast cell hematopoietic malignancy) 2. Palpable hepatomegaly with ascites, impairment of liver function, and/or portal hypertension 3. Palpable splenomegaly with hypersplenism 4. Bone/skeletal findings demonstrating large osteolytic lesions (≥2 cm) with pathologic fracture ± bone pain 5. Malabsorption with weight loss due to gastrointestinal mast cell infiltrates
SM-AHN	SM with criteria for a non–mast cell hematologic syndrome or neoplasia based on the WHO criteria. Of these patients, 90% have a hematologic malignancy that is myeloid in origin (eg, a myeloproliferative neoplasm or myelodysplastic syndrome). However, there are rarer instances of leukemic transformation, whereby patients present with acute myeloid leukemia or a lymphoproliferative disorder (such as multiple myeloma, Hodgkin lymphoma, or non-Hodgkin lymphoma)
MCL	SM that is often initially indistinguishable from ISM; however, during the natural course of the disease, patients will have a bone marrow biopsy sample infiltrated by a large number of atypical mast cells (≥20% of the nucleated cells in the bone marrow aspirate). These cells are often immature and hypogranulated with a blast-like morphology (high nucleus-to-cytoplasm ratios and presence of mitotic figures and multilobed nuclei)

Solid mast cell tumors, including mast cell sarcoma and mastocytoma, are not considered forms of SM; therefore, patients with such tumors were not included in our cohort.

### Delay in SM diagnosis

The complete EMRs of all patients with confirmed SM were thoroughly reviewed, and the earliest time point of mast cell–related symptoms was determined. If symptoms were directly related to eventual SM diagnosis, the time at which the patient reported symptom initiation was utilized (and confirmed by EMRs, whenever possible). Symptoms included abdominal pain, allergic reaction, anaphylaxis, angioedema, diarrhea, hives, rash, and urticaria*,* and any “B” or “C” finding on laboratory test or imaging results. Delay in diagnosis was determined on the basis of time between the date of the initial presenting symptoms and the date of definitive diagnosis.

### SM progression and all-cause mortality

Patients with confirmed SM were grouped by SM subtype (Table I), and the progression status and interval from ISM to advanced SM was determined. Time to progression was determined by calculating the interval in months between time of definitive diagnosis to time of first presentation of either 2 “B” findings, at least 1 “C” finding, or other evidence of either an associated hematologic malignancy or MCL. For both SM progression and mortality analyses, the end of follow-up date was the date of death, study end date (September 30, 2022), or date on which the patient was lost to follow-up (no longer enrolled at KPSC).

### Patient characteristics

Patient demographics, lifestyle characteristics, and health plan enrollment and coverage information were extracted from the KPSC Research Data Warehouse.^10^ The Neighborhood Deprivation Index, a community-level measure assessing neighborhood socioeconomic context, was also included in the analysis.^11^^,^^12^

### Health care resource utilization

Health care resource utilization, including hospital admission, emergency department visits, urgent or nonurgent care visits, referrals, and treatment of patients with SM, was calculated by dividing the total number of events by the total observation time (health plan enrollment period) and reported as rate per person per year. Details of how medication dispensing events were recorded are listed in the Methods section of the Online Repository.

### Statistical analysis

All of the analyses were descriptive. Patient characteristics, overall and by subtype of SM, are presented as numbers and percentages, means and SDs, and medians and interquartile ranges (IQRs). For health care resource utilization, including treatment of patients with SM, we reported the number of events and the rate (per person per year). Kaplan-Meier curves were used to plot the cumulative progression and the survival curves across an average follow-up period of 74.2 months (range 2-295 months, median 55 months).

## Results

### Eligible patients with SM

In total, 116 patients met the 2016 WHO criteria for SM. The median years of total health plan enrollment was 6.7 (IQR = 5.4-11.4). The median years of health plan enrollment before and after the first SM diagnosis at KPSC were 5.0 (IQR = 1.8-5.0) and 3.5 (IQR = 1.2-7.3), respectively. In all, 94 patients met at least 1 major criterion (focal aggregates of >15 mast cells per cluster) and at least 1 minor criterion, and 13 patients met at least 3 minor criteria (Table II). Of the patients who met minor criteria, 67% (n = 78) had more than 25% spindle-shaped mast cells; 36% (n = 42) had a recorded *D816V* mutation; 82% (n = 95) had a tryptase level higher than 20 ng/mL; and 72% (n = 85) had expression of CD2, CD25, or CD30 on mast cell staining (Table II). The remaining 9 patients, who did not strictly meet the WHO criteria on the basis of the available data (see the Methods section of the Online Repository), were nevertheless still included in our SM cohort if they could be manually confirmed as having SM on the basis of patient and clinical reports in the EMR. That is, in some patient records the progress notes from treating physicians were clear enough to strongly suspect that the patient met the criteria for SM before their enrollment in KPSC (some were even treated for SM with SM-specific US Food and Drug Administration–approved cytoreductive therapies), and several were known to have been diagnosed at well-respected academic institutions (even though specific biopsy reports and other clinical findings were not always provided by the patients or their previous physicians).

**Table II:** Identification of the cohort of patients with SM (according to the 2016 WHO criteria)

SM assessment	No. of manually confirmed patients with SM (N = 116)
SM cohort	
Criteria met: 1 major + ≥1 minor	94 (80.0%)
Criteria met: ≥3 minor	13 (11.2%)
Criteria not met, but SM was clinically confirmed∗	9 (7.8%)
Minor criteria among the patients with SM	
Spindle-shape cells	78 (67.2%)
*c-KIT*–positive	42 (36.2%)
Tryptase- positive	95 (81.9%)
CD2, CD25, and/or CD30-positive	85 (72.3%)
Biopsy performed	
Bone marrow	114 (98.3%)
Skin	29 (24.8%)
Colonoscopy	14 (11.9%)

∗ Based on convincing historical data, including partial bone marrow reports, laboratory test values, past treatments, and available physician notes.

In all, 98.3% of patients (n = 114) received a confirmatory bone marrow biopsy as part of their SM workup, whereas 11.9% of patients (n = 14) had a confirmatory colonoscopy biopsy (Table II). Of the 116 patients with SM, 24.8% (n = 29) underwent a skin biopsy as part of the workup for diagnosing cutaneous mastocytosis during their disease workup. Interestingly, 3 patients were never diagnosed as having SM yet were captured on the basis of the review of their bone marrow biopsy reports.

### SM subtypes

Among the 116 patients with confirmed SM, the subtypes identified at initial diagnosis were as follows: indolent SM, 77% (n = 89); smoldering SM (SSM), 2% (n = 2); SM-AHN, 12% (n = 14); ASM, 9% (n = 11); and MCL, 0% (n = 0) (Table III).

**Table III:** Diagnostic characteristics in all patients manually confirmed as having SM, by SM subtype at initial diagnosis

Diagnostic characteristics	SM subtype				Total (N = 116)
	ISM (n = 89)	SSM (n = 2)	SM-AHN (n = 14)	ASM (n = 11)	
Specialty of provider who made the first EMR-based SM diagnosis at KPSC, no. (%)∗					
Internal/family medicine	19 (21.4%)	1 (50.0%)	2 (14.3%)	2 (18.2%)	24 (20.7%)
Hematology/oncology	41 (46.1%)	1 (50.0%)	12 (85.7%)	8 (72.7%)	62 (53.4%)
Allergy and immunology	8 (8.9%)	0	0	1 (9.1%)	9 (7.8%)
Gastroenterology	9 (10.1%)	0	0	0	9 (7.8%)
Dermatology	9 (10.1%)	0	0	0	9 (7.8%)
Unknown	3 (3.4%)	0	0	0	3 (2.6%)
Time from month of EMR diagnosis to month of definitive diagnosis†					
Mean (SD)	–1.5 (42.1)	–1	–1.1 (2.1)	0.1 (6.7)	–1.2 (36.9)
Median (IQR)	0 (–1 to 0)	–1 (–1 to –1)	0 (–1 to 0)	–1 (–1 to 0)	0 (–1 to 0)
Comparison of month of definitive diagnosis and month of EMR-based diagnosis, no. (%)†					
Month of definitive diagnosis was before the month of EMR-based diagnosis	34 (44.7%)	1 (100%)	5 (45.5%)	7 (63.7%)	47 (47.5%)
Month of definitive diagnosis was the same as the month of EMR-based diagnosis	24 (31.6%)	0	6 (54.5%)	1 (9.1%)	33 (33.3%)
Month of definitive diagnosis was after the month of EMR-based diagnosis	18 (23.7%)	0	0	3 (27.3%)	19 (19.2%)
Time from month of symptom presentation to the month of definitive diagnosis†					
Mean (SD)	66.9 (32.5)	72	26.6 (43.3)	28.8 (24.9)	58.3 (73.1)
Median (IQR)	32.5 (6-120)	72 (72-72)	4.0 (1-37)	29 (3-45)	29.0 (4-96)
Comparison of month of definitive diagnosis and month of symptom presentation, no. (%)†					
Symptom presentation month was before the month of definitive diagnosis	66 (86.8%)	1 (100%)	9 (81.8%)	11 (100%)	87 (87.9%)
Month of symptom presentation was the same as month of definitive diagnosis	10 (13.2%)	0	2 (18.2%)	0	12 (12.1%)

∗ Of the 24 patients who were first diagnosed by internal/family medicine physicians, 1 was diagnosed before joining KPSC.

† In patients with SM who were first diagnosed at KPSC or whose information before joining KPSC was documented during the period covered by the EMR. A negative number indicates that the month of the definitive diagnosis was before the month of the EMR-based diagnosis. Calculations for this entry/series of entries were based on the following sample sizes: 76 patients with ISM, 1 patient with SSM, 11 patients with SM-AHN, 11 patients with ASM, and 99 patients in total.

### Delay in SM diagnosis

The average delay to diagnosis of SM (from the time of initial presentation of signs and/or symptoms suspicious for SM) was 58.3 months (Table III). In general, the advanced forms of SM had a shorter delay to diagnosis time than the nonadvanced types (ISM and SSM), underscoring the difficulty in identifying subtle forms of the disease. Additionally, 5 patients who presented with an advanced form of SM were initially misdiagnosed as having only ISM or SSM, whereas 3 patients were never formally diagnosed with any form of SM despite meeting the WHO criteria (missed diagnosis).

The diagnosis of SM was made most frequently by practitioners in the following specialties: hematology/oncology (n = 62), followed by allergy/dermatology/gastrointestinal diseases (n = 27 [9 in each specialty]), and internal/family medicine (n = 24) (Table III). All subtypes showed a predominance toward non-Hispanic White populations, followed by a sizeable Hispanic patient population, and then Black patients and Asian/Pacific Islander patients (Table IV). The mean score for the Neighborhood Deprivation Index was generally similar across the different SM subtypes.

**Table IV:** Patient and provider characteristics by subtypes of SM

Patient or provider characteristics	SM subtype				All (N = 116)
	ISM (n = 89)	SSM (n = 2)	SM-AHN (n = 14)	ASM (n = 11)	
Age (y)					
Mean (SD)	52.8 (14.1)	71 (4.2)	67 (14.3)	63.5 (15.3)	55.9 (15.1)
Median (IQR)	53 (43-64)	71 (68-74)	69 (58-77)	63 (54-77)	56.5 (44.5-68)
Sex, no. (%)					
Male	37 (41.6%)	2 (100%)	11 (78.6%)	9 (81.8%)	59 (50.9%)
Female	52 (58.4%)	0	3 (21.4%)	2 (18.2%)	57 (49.1%)
Race/ethnicity, no. (%)					
Non-Hispanic White	55 (61.8%)	1 (50.0%)	6 (42.9%)	6 (54.5%)	68 (58.6%)
Hispanic	20 (22.5%)	0	6 (42.9%)	3 (27.3%)	29 (25.0%)
Black	3 (3.4%)	1 (50.0%)	0	2 (18.2%)	6 (5.2%)
Asian/Pacific Islander	6 (6.7%)	0	2 (14.3%)	0	8 (6.9%)
Other	5 (5.6%)	0	0	0	5 (4.3%)
Length of health plan enrollment (y)					
Mean (SD)	14.5 (13.9)	23.8 (18.1)	18.0 (16.3)	21.0 (22.3)	15.7 (15.1)
Median (IQR)	9.7 (4.4-19.2)	23.8 (11.0-36.6)	13.7 (8.3-21.2)	9.2 (4.5-44.7)	10.5 (5.1-21.2)
Never enrolled or enrolled after first SM diagnosis, no. (%)	2 (2.3%)	0	1 (7.1%)	0	3 (2.6%)
Insurance type, no. (%)					
Commercial	58 (65.2%)	1 (50.0%)	4 (28.6%)	4 (36.4%)	67 (57.8%)
Medi-Cal/other state programs	5 (5.6%)	0	1 (6.7%)	3 (27.3%)	9 (7.7%)
Medicare	20 (22.5%)	1 (50.0%)	8 (53.3%)	6 (54.6%)	35 (29.9%)
Private pay	21 (23.6%)	1 (50.0%)	6 (40.0%)	5 (45.5%)	33 (28.2%)
BMI, m/kg^2^					
Mean (SD)	28.7 (5.6)	23.8 (1.4)	24.5 (3.8)	25.6 (3.4)	27.9 (5.4)
Normal, no. (%)	20 (22.5%)	2 (100%)	7 (50.0%)	6 (54.6%)	35 (30.2%)
Overweight, no. (%)	34 (38.2%)	0	5 (35.7%)	2 (18.2%)	41 (35.3%)
Obese, no. (%)	28 (31.5%)	0	1 (7.1%)	1 (9.0%)	30 (25.9%)
Unknown, no. (%)	7 (7.8%)	0	1 (7.1%)	2 (18.2%)	10 (8.6%)
Exercise (min/wk)					
Exercised, no. (%)	36 (40.5%)	0	9 (64.3%)	3 (27.3%)	48 (41.4%)
No exercise, no. (%)	23 (25.8%)	1 (50.0%)	3 (21.4%)	4 (36.4%)	31 (26.7%)
Unknown, no. (%)	30 (33.7%)	1 (50.0%)	2 (14.3%)	4 (36.4%)	37 (31.9%)
Minutes/wk, mean (SD)	137.9 (162.3)	0	120 (108.1)	50 (79.4)	125.7 (150.2)
Median (IQR)	90 (0-240)	0	110 (0-210)	0 (0-150)	60 (0-210)
Smoking status, no. (%)					
Yes	5 (5.6%)	0	0	0	5 (4.3%)
Never	53 (59.6%)	1 (50.0%)	8 (57.1%)	7 (63.6%)	69 (59.5%)
Quit	18 (20.2%)	1 (50.0%)	5 (35.7%)	3 (27.3%)	27 (23.3%)
Passive	1 (1.1%)	0	0	0	1 (0.9%)
Unknown	12 (13.5%)	0	1 (7.1%)	1 (9.1%)	14 (12.1%)
Neighborhood Deprivation Index					
Mean (SD)	0.4 (0.3)	0.5 (0.3)	0.5 (0.2)	0.5 (0.3)	0.4 (0.3)

### SM progression and all-cause mortality

As already stated in the Methods section, analysis of progression and all-cause mortality was performed on patients for whom sufficient historical data were available, such that a definitive date of diagnosis or date of earliest EMR coding could be verified. Of the patients who had sufficient historical data and were initially diagnosed as having nonadvanced forms of SM (ie, ISM or SSM [n = 87]), 18% (n = 16) progressed to a more advanced form of SM (from ISM to ASM, n = 6; from ISM to SM-AHN, n = 8; from SSM to SM-AHN, n = 1; and from ISM to MCL, n = 1 [data not shown]). The average time of progression from nonadvanced forms of SM to advanced forms of SM (SM-AHN, ASM, or MCL) was 88.3 months (range 2-295). Progression from ISM/SSM to SM-AHN averaged 54.1 months (median 23 months, range 2-159 months). This included 2 patients who experienced a leukemic transformation (to acute myeloid leukemia), whereas others experienced myeloproliferative disorders (n = 5) or multiple myeloma (n = 1). Time to progression to ASM averaged 124.2 months (median 117 months; range 13-295 months). Finally, the 1 patient who progressed to MCL did so over the course of 15 years (180 months).

**Fig 1:**
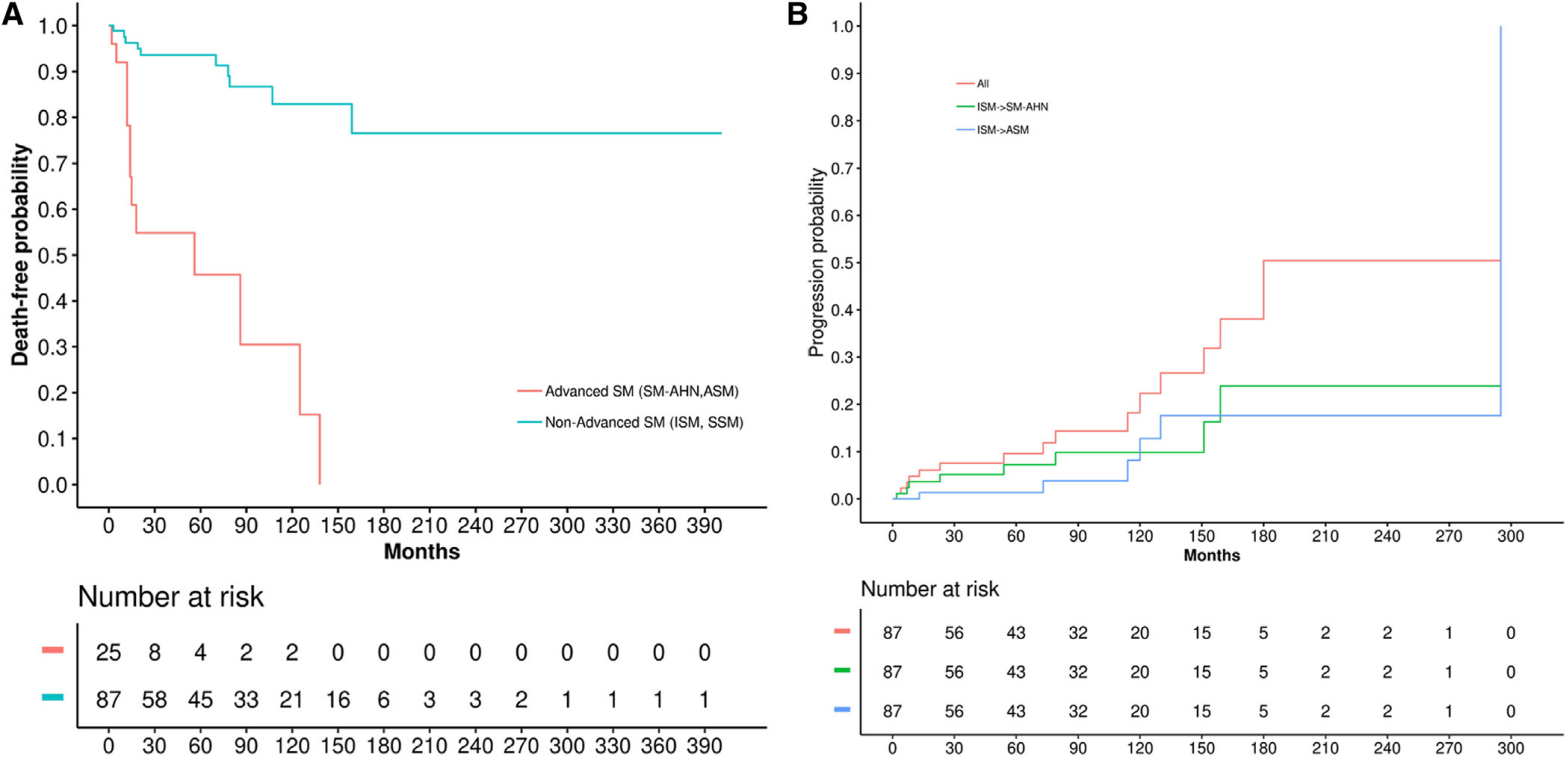
**A,** Kaplan-Meier curve for SM progression from nonadvanced SM to any advanced SM. Nonadvanced SM includes ISM and SSM, whereas advanced SM includes SM-AHN, ASM, and MCL. **B,** Kaplan-Meier curve for death from SM diagnosis to death.

Survival curves demonstrated that there is a definite increase in the probability of mortality once a patient has been diagnosed with an advanced SM subtype of any kind (SM-AHN, ASM, or MCL) (Fig 1).

### Health care resource utilization

On average, patients with SM had increases in health care utilization after diagnosis versus before diagnosis. This includes increases in hospital admissions, ER visits, urgent care visits, and specialty provider visits. The greatest increase in specialist visits was noted in hematology/oncology, followed by allergy/immunology, dermatology, and gastroenterology. Similarly, certain mast cell–related treatments saw an increase in use after SM diagnosis—in particular, with respect to epinephrine autoinjectors, systemic corticosteroids, H1/H2 antihistamines, leukotriene modifiers, and cromolyn dispensed (Table V). After diagnosis of SM, cytoreductive therapies used to treat SM saw an increase in use as well (Table V).

**Table V:** Health care resource utilization before and after SM diagnosis

Health care resource	Before SM diagnosis		After SM diagnosis	
	No. of events∗	Rate/y (95% CI)†	No. of events∗	Rate/y (95% CI)‡
Hospital admission	53	0.1 (0.1-0.2)	159	0.3 (0.2-0.3)
Emergency department visit	136	0.3 (0.3-0.4)	282	0.5 (0.4-0.6)
Urgent care	126	0.3 (0.3-0.4)	240	0.4 (0.4-0.5)
Nonurgent care clinic/virtual visits	4618	11.4 (11.1-11.7)	9471	16.9 (16.5-17.2)
Specialty visit				
Hematology/oncology	670	1.7 (1.5-1.8)	4114	7.3 (7.1-7.5)
Allergy and immunology	222	0.5 (0.5-0.6)	939	1.7 (1.6-1.8)
Gastroenterology	328	0.8 (0.7-0.9)	636	1.1 (1.0-1.2)
Dermatology	528	1.3 (1.2-1.4)	882	1.6 (1.5-1.7)
Tryptase test	101	0.2 (0.2-0.3)	849	1.5 (1.4-1.6)
Service referred for or advice requested for				
Allergy	28	0.07 (0.05-0.1)	42	0.07 (0.05-0.1)
Dermatology	59	0.1 (0.1-0.2)	51	0.09 (0.07-0.1)
Gastroenterology	62	0.2 (0.1-0.2)	94	0.2 (0.1-0.2)
Hematology	92	0.2 (0.2-0.3)	54	0.1 (0.07-0.1)
Treatment				
EPIPEN autoinjector	33	0.08 (0.06-0.1)	152	0.3 (0.2-0.3)
Epinephrine vial	0	0	12	0.02 (0.01-0.04)
Systemic steroid	100	0.2 (0.2-0.3)	514	0.9 (0.8-1.0)
Inhaled corticosteroid	49	0.1 (0.09-0.2)	98	0.2 (0.1-0.2)
H1 antihistamine	114	0.3 (0.2-0.3)	447	0.8 (0.7-0.9)
H2 antihistamine	83	0.2 (0.2-0.3)	615	1.1 (1.0-1.2)
Leukotriene receptor antagonists and 5-lipoxygenase inhibitor	45	0.1 (0.08-0.1)	296	0.5 (0.5-0.6)
Cromolyn sodium	2	0.005 (0.001-0.02)	127	0.2 (0.2-0.3)
Chemotherapy or targeted agent for SM	0	0	450	0.8 (0.7-0.9)
Osteoporosis drug	122	0.3 (0.3-0.4)	91	0.2 (0.1-0.2)
Biologic§	0	0	0	0
Proton pump inhibitor	130	0.3 (0.3-0.4)	434	0.8 (0.7-0.8)

∗ Number of events, including number of records for dispensing of outpatient prescriptions and number of inpatient and/or emergency room admissions with dispensed medications.

† Total follow-up time before diagnosis date, 405.9 person years.

‡ Total follow up time after diagnosis date, 561.8 person-years.

§ Includes benralizumab, dupilumab, mepolizumab, omalizumab, reslizumab, and tezepelumab.

## Discussion

The 116 patients with confirmed SM who were identified in a large health maintenance organization provided a unique opportunity to describe various important features of the natural history of SM, including disease progression, delay to diagnosis, and health care utilization. Consistent with previously published data, White individuals comprise the majority of the patients with SM in our cohort,^13^, ^14^, ^15^, ^16^ but our study also found that Hispanic individuals comprise a significant portion of patients with SM as well. Although this finding likely reflects our Southern California population, it highlights the need for additional studies in non-White, and non-European populations—groups that are often understudied, perhaps owing to disparities in access to health care.

Also of particular interest was the finding that there was a delay to diagnosis of SM (by a mean of 58.3 months) from time of symptom presentation to definitive diagnosis (meeting the WHO criteria). Prior publications have reported a similar time frame of delay to diagnosis,^17^ but we believe that ours is the first study to use patient EMRs (as opposed to patient-reported surveys, which are susceptible to recall bias) to define the start of SM symptoms, and also to track the records until a definitive diagnosis has finally been made. This finding presents physicians with a window of opportunity to intervene in the disease process earlier, and with the recent advancements with regard to treatment options for both nonadvanced and advanced forms of SM, it is imperative that the physician community (including primary care physicians, who were the first to code for SM in 20% of our cohort) be more keenly aware of these patients’ signs and symptoms and order the necessary referrals and laboratory and other diagnostic tests (eg, testing for *c-KIT* mutational status was not even ordered for 23 patients). When this is done, these patients can be referred to specialists more quickly, and their disease burden can be lessened. It is reasonable to postulate that as the medical community becomes more familiar with the signs and symptoms of nonadvanced subtypes of SM (and its treatments), a shorter time to definitive diagnosis of SM may result in reduced health care utilization overall (emergency room visits, hospitalizations, urgent and nonurgent clinic visits and procedures, and polypharmacy). Other important findings in this study include the finding that treatments targeted toward SM increase in frequency of use once the diagnosis of SM has been made.

Although our study showed that hematologists definitively diagnosed SM more frequently than other specialists did, the findings of other studies differed.^17^^,^^18^ This may reflect the unique referral practices at each institution. But it may also have to do with different study designs. For example, 2 of the studies indicating either dermatology^18^ or allergy^17^ as the most frequent specialties to diagnose SM were both done by using patient surveys, which may be subject to recall bias, whereas our study was done by physician review of EMRs. Some studies also did not specify SM (asking instead about physician-diagnosed “mastocytosis”), and because many more patients with mastocytosis actually have cutaneous mastocytosis (rather than SM), this likely led to the higher frequency of cases of dermatologist-diagnosed “mastocytosis.”

Another interesting finding is the low prevalence of the *D816V* mutation in our SM population (ie, of the 93 patients tested for their mutation status, 42 were found to have *c-KIT* *D816V* mutations, which equates to about a 45% positivity rate for those tested for the *c-KIT* *D816V* mutation). It was noted, however, that nearly all patients had *D816V* mutational analyses performed by using older testing methods (that can miss 72% of patients with the mutation),^7^ which were less sensitive^13^ than the testing that is available today.^19^ It is possible that rerunning these patient samples using newer testing methods would yield a higher rate of *c-KIT* *D816V* mutations. Furthermore, rarely were *c-KIT* mutations other than *D816V* ever checked.

It is worth noting that our prevalence data resemble the data regarding other cohorts,^20^, ^21^, ^22^ in which patients with nonadvanced SM make up the majority of their SM population and ASM, SM-AHN, and MCL are similarly represented. Our cohort had a somewhat smaller percentage of patients with SM-AHN than those in some studies with a longer follow-up period^20^ (thus, providing more time for patients to progress to more advanced subtypes). Also, regarding disease progression (16 of our patients [18%] progressed from nonadvanced SM to a more advanced subtype), the rate of progression (shown in Fig 1) was found to be similar^23^ or somewhat higher (roughly 10%-15% higher) than the rates in previously published progression data,^24^, ^25^, ^26^ perhaps owing to our longer follow-up time and to the KPSC health system (in which “full capture” of data is possible). Finally, our survival curves (shown in Fig 1) mirror those of these previous publications.^21^^,^^26^^,^^27^

This retrospective study has certain limitations. For example, some patients had a prior diagnosis of SM, and a subset of those patients had only incomplete diagnostic data and clinical history. Also, cumulative risk analyses and survival curves were affected not only by clinical events such as death but also by the date on which a patient disenrolled at KPSC (such that both death and disenrollment affected the calculated mortality rate at any given time point). As such, it is believed that our risk of death is probably an overestimation.

In summary, patients with SM have a lengthy delay to definitive diagnosis and a high level of health care utilization after diagnosis, and although most patients with nonadvanced SM remain in that state, there is still an 18% rate of progression to more advanced subtypes of SM. With the availability of high-sensitivity *c-KIT* *D816V* mutational analysis of both peripheral blood and bone marrow (performed by droplet digital PCR) and effective (US Food and Drug Administration–approved) treatments for both ISM (avapritinib) and advanced SM subtypes (avapritinib, midostaurin, and imatinib) available on the US market, it is hoped that in this new era of SM diagnosis and treatment, substantial strides will be made in the areas of improving overall patient care, quality of life, and health care utilization.

## Disclosure statement

Supported by Blueprint Medicines (Cambridge, Mass) through an unrestricted grant to Kaiser Permanente Southern California, as well as by the National Heart, Lung and Blood Institute (grant to R.S.Z.)

Disclosure of potential conflict of interest: for Kaiser Permanente Southern California: R. S. Zeiger has received grants from Aerocrine, 10.13039/100004328Genentech, 10.13039/501100004628MedImmune/10.13039/100004325AstraZeneca, Merck, 10.13039/100004330GlaxoSmithKline, Teva, and Quest; has warrants from DBV Technologies; and has provided consultant activity for AstraZeneca, Bayer, Regeneron/Sanofi, Merck, and Genentech/Novartis. K. Miller, D. Powell, B. Lampson, C. Yuen, D. Cattie, T. Green, and E. Sullivan are employed by Blueprint Medicines. The rest of the authors declare that they have no relevant conflicts of interest.

## Supplementary data

Supplementary Tables

Supplementary Figure E1
