# Supplementary material for: MASTering systemic mastocytosis: Lessons learned from a large patient cohort
Source: J Allergy Clin Immunol Glob. 2024 Jul 27;3(4):100316. doi: 10.1016/j.jacig.2024.100316 (PMC11372574; doi:10.1016/j.jacig.2024.100316)
Supplement: Supplementary Appendix [file mmc1.docx]

**APPENDIX**

**MASTering Systemic Mastocytosis: Lessons learned from a large patient cohort**

**Kevin Y. Tse, MD, MS, Wansu Chen, MS, PhD, Eric J. Puttock, PhD, Shanta Chowdhury, MS, PhD, Kerri Miller, PharmD, Dakota Powell, MPH, Benjamin. Lampson, MD, PhD, Chris Yuen, PharmD, Doug Cattie, PhD, Teresa Green, MSPH, Erin Sullivan, PhD, MPH, Robert S. Zeiger MD, PhD**

**METHODS:**

*Identification of eligible SM patients and their subtypes*

The ICD-9 and ICD-10 codes used to screen for patients who had been diagnosed with SM are shown in Table E2. Nine patients were still confirmed to have SM despite having only partially complete data, but sufficient historical data to support SM diagnosis. These data include specialists’ notes, frequency/persistence of SM diagnosis codes, prior lab values suggestive of SM (elevated tryptase or presence of c-KIT D816V mutation, any available bone marrow biopsy reports), and receiving treatments specific for SM (i.e. mast cell specific cytoreductive therapies). These patients all met less than or equal to 2 minor criteria (n=8) or only one major criterion without minor criteria (n=1). These “historically” diagnosed patients (who did not fulfill all of the WHO 2016 criteria), were all patients who had been diagnosed with SM prior to enrolling with KPSC. There were also 3 subjects who met WHO criteria but were never formally diagnosed with SM. These patients had bone marrow (or other extra-cutaneous) biopsy evidence of SM, but clinicians did not recognize the available data. They were identified by using keyword search “mast cell” in bone marrow, intestinal, or skin biopsy reports, and then confirmed by manual review (See Supplemental Figure E1).

*Delay in SM diagnosis*

Symptoms were manually chart-reviewed, and any symptoms that lead to the eventual diagnosis of SM (via specialty referral, lab/imaging study performed, or biopsies done) were time stamped. Symptom initiation often preceded the definitive diagnosis (the time at which the patient met the WHO criteria), as these symptoms were the presenting symptoms at their doctors’ office visits. For those patients whose historical data was limited, and if a definitive time point of initial presentation could not be determined via chart review, they were not included in the time to delay in diagnosis analysis. Some patients had a diagnosis of SM coded in the EMR before their definitive diagnosis date, presumably because symptoms were so compelling that the physician felt confident coding SM before the workup was complete. We used the earlier of the two dates (documented symptom start date, and the documented EMR-coded date) to perform the progression and mortality analysis but applied the SM coding date to conduct the other analyses (i.e., patients characteristics and healthcare resource utilization).

*SM progression and all-cause mortality*

In cases where a physician coded SM in the EMR on a date prior to the definitive diagnosis, we used the EMR date as the starting date for our calculation. For those patients who had only limited historical data (n=2), or they were not a KPSC enrollee when they were first diagnosed at KPSC (n=2), they were excluded in the all-cause mortality analysis. An additional 25 patients (11 ASM and 14 SM-AHN) were excluded from the SM progression analysis because the patients already met the definition for advanced SM at the time of initial diagnosis at KPSC.

*Healthcare resource utilization*

For outpatient medication dispensing, an “event” was defined as one dispensing record; however, for inpatient admissions or ED visits, an “event” referred to an encounter regardless of the number of medication administration during the hospital stay or ED visit. Epinephrine vials were limited to usage related to allergy or anaphylaxis. For chemo or targeted agents for SM treatment, only the time period the medication was available to KPSC patients was considered the “at-risk” time and was considered in the calculation of the denominators.
