# Supplementary material for: MASTering systemic mastocytosis: Lessons learned from a large patient cohort
Source: J Allergy Clin Immunol Glob. 2024 Jul 27;3(4):100316. doi: 10.1016/j.jacig.2024.100316 (PMC11372574; doi:10.1016/j.jacig.2024.100316)
Supplement: Supplementary Tables [file mmc2.docx]

**Table E1. Number and cause of death by SM type (non-advanced vs. advanced).**

| **Cause of death categories based on ICD-10 codes*** | **Non-advanced SM**  **N=10** |  | **Advanced SM**  **N=13** |
| --- | --- | --- | --- |
| Neoplasms (C00-D48) | 2 |  | 4 |
| Diseases of the circulatory system (I00-I99) | 2 |  | 1 |
| Endocrine, nutritional and metabolic diseases (E00-E88) | 2 |  | 0 |
| Diseases of the genitourinary system (N00-N98) | 0 |  | 2 |
| Diseases of the blood and blood-forming organs and certain disorders involving the immune mechanism (D50-D89) | 0 |  | 1 |
| Diseases of the nervous system (G00-G98) | 1 |  | 0 |
| Diseases of the respiratory system (J00-J98) | 0 |  | 1 |
| Diseases of the digestive system (K00-K92) | 1 |  | 0 |
| Congenital malformations, deformations and chromosomal abnormalities (Q00-Q99) | 0 |  | 1 |
| Unknown | 2 |  | 2 |

**Table E2. ICD codes used to identify SM patients.**

| ICD_code type | ICD code |
| --- | --- |
| ICD9 | 202.60, 202.61, 202.62, 202.63, 202.64, 202.65, 202.66, 202.67, 202.68, 207.80, 207.81, 207.82 |
| ICD10 | C94.30, C94.31, C94.32, C96.21, D47.02 |

**Table E3. Patients diagnosed with SM but were not confirmed based on WHO 2016 criteria (n=298).**

| Group name | Number of Patients |
| --- | --- |
| r/o SM by BM biopsy reports (with or without minor criteria) -- > NO | 117 |
| No BM biopsy report found but met 1 or 2 minor criteria -- > likely | 17 |
| No BM biopsy report found and did not meet any minor criterion -- > not likely | 164 |

**Table E4. Patient and provider characteristics by advanced and non-advanced SM subtype.**

| Patient or provider characteristics | Non-Advanced SM  [ISM, SSM]  (N=91) | Advanced SM  [SM-AHN, ASM]  (N=25) |
| --- | --- | --- |
| Age |  |  |
| Mean (SD) | 53.2 (14.2) | 65.4 (14.5) |
| Median (IQR) | 53 (43-65) | 66 (58-77) |
| Sex |  |  |
| Male, (N, %) | 39 (42.9) | 20 (80.0) |
| Female, (N, %) | 52 (57.1) | 5 (20.0) |
| Race/ethnicity |  |  |
| Non-Hispanic White, (N, %) | 56 (61.5) | 12 (48.0) |
| Hispanic, (N, %) | 20 (22.0) | 9 (36.0) |
| Black, (N, %) | 4 (4.4) | 2 (8.0) |
| Asian/Pacific Islander, (N, %) | 6 (6.6) | 2 (8.0) |
| Others/Unknown, (N, %) | 5 (5.5) | 0 |
| Length of health plan enrollment | 14.7 (13.9) | 18.8 (18.7) |
| BMI |  |  |
| Mean (SD) | 28.6 (5.6) | 25.0 (3.6) |
| Normal, (N, %) | 22 (24.1) | 13 (52.0) |
| Overweight, (N, %) | 34 (37.4) | 7 (28.0) |
| Obese, (N, %) | 28 (30.8) | 2 (18.0) |
| Unknown, (N, %) | 7 (7.7) | 3 (12.0) |
| Exercise minute per week |  |  |
| Exercised, (N, %) | 36 (39.6) | 12 (48.0) |
| No exercise, (N, %) | 24 (26.4) | 7 (28.0) |
| Unknown, (N, %) | 31 (34.0) | 6 (24.0) |
| Minutes/wk, mean (SD) | 135.7 (161.9) | 94.2 (102.2) |
| Median (IQR) | 75 (0-225) | 60 (0-180) |
| Neighborhood Deprivation Index (NDI) |  |  |
| Mean (SD) | 0.4 (0.3) | 0.5 (0.2) |
| Smoking status |  |  |
| Yes, (N, %) | 5 (5.5) | 0 |
| Never, (N, %) | 54 (59.3) | 15 (60.0) |
| Quit, (N, %) | 19 (20.9) | 8 (32.0) |
| Passive, (N, %) | 1 (1.1) | 0 |
| Unknown, (N, %) | 12 (13.2) | 2 (8.0) |
| Insurance types |  |  |
| Commercial, (N, %) | 59 (64.8) | 8 (32.0) |
| Medi-Cal/other State programs, (N, %) | 5 (5.5) | 4 (16.0) |
| Medicare, (N, %) | 21 (23.1) | 14 (56.0) |
| Private pay, (N, %) | 22 (24.2) | 11 (44.0) |
| Never enrolled or enrolled after 1st SM diagnosis, (N, %) | 2 (2.2) | 1 (4.0) |
