# Supplementary material for: MASTering systemic mastocytosis: Lessons learned from a large patient cohort
Source: J Allergy Clin Immunol Glob. 2024 Jul 27;3(4):100316. doi: 10.1016/j.jacig.2024.100316 (PMC11372574; doi:10.1016/j.jacig.2024.100316)
Supplement: Supplementary Figure E1 [file mmc3.docx]

**Figure E1**
